# Sexual decision-making: an exploratory interview study of Cambodian adolescents

**DOI:** 10.3389/frph.2024.1409351

**Published:** 2024-12-02

**Authors:** Gloria Park, Youngran Yang

**Affiliations:** ^1^ St. David’s School of Nursing, Texas State University, Round Rock, TX, United States; ^2^ School of Nursing, Research Institute of Nursing Science, Sustainable Development Center, Jeonbuk National University, Jeonju, Republic of Korea

**Keywords:** decision-making, adolescents, sex, Qualitative Research, Cambodia, interview, internal factors, external factors

## Abstract

**Introduction:**

The rate of sexual activity among adolescents is very high, with serious repercussions such as human immunodeficiency virus (HIV) and sexually transmitted diseases. Understanding the factors that influence adolescents’ engagement in sexual activity is crucial for promoting healthy sexual attitudes and behaviors in schools, sex education programs, communities, and families. This study aimed to examine the factors influencing sexual decision-making among Cambodian adolescents.

**Methods:**

In accordance with the Standards for Reporting Qualitative Research (SRQR), this study used a descriptive qualitative methodology with individual interviews. The participants in the study were 30 Cambodian adolescents (15 males and 15 females) who were all unmarried and sexually active. They were recruited using various methods, including social networking services, and interviewed to explore their sexual decision-making processes.

**Results:**

The analysis revealed that the decision-making process was influenced by both internal and external factors. Internal factors included sexually explicit Internet material and arousal from sexy outfits, while external factors included foreign vs. Khmer culture, the surrounding environment including community, peers, and family, and educational advice received at school. Gender differences were noted in responses to stimuli like sexy outfits and perceptions of cultural norms.

**Conclusions:**

This study underscores the complexity of adolescent sexual decision-making in Cambodia. It highlights the need for sex education that is not only comprehensive but also culturally sensitive, addressing the diverse influences on these adolescents. Future research should include a broader demographic group, including rural adolescents, to gain more comprehensive insights.

**Implications for practice:**

This study uncovers how cultural norms, peers, and the media impact sexual behaviors, emphasizing the significant gender differences in these aspects. The findings shed light on the necessity of culturally sensitive and comprehensive sex education and the urgent need for tailored approaches to health promotion and education.

## 1 Introduction

Adolescence is a critical period for sexual and reproductive health as sexual curiosity and intimacy increase dramatically (1). The prevalence of sexual intercourse among young adolescents aged 12–15 years ranges from 1.2% (Western Pacific region) to 17.8% (American region) worldwide (1). However, its prevalence is much higher among older adolescents. Studies reported that more than half of adolescents engaged in sexual activities, including penile-vaginal intercourse or oral sex among adolescents aged 15–19, in the United States (2), 18.1% in China (3), and up to 58.6% in Caribbean countries (4). A recent survey in Cambodia reported that 16% of females initiated sexual intercourse by the age of 18, and this rate was higher among women with no education or low economic status (5).

Early sexual engagement may result in poor sexual and reproductive health outcomes, such as unintended pregnancy, sexually transmitted infections (STIs) including human immunodeficiency virus (HIV), and mental health issues among adolescents (1). The factors that influence early sexual initiation among adolescents are diverse. For instance, in Bangladesh, illiteracy, primary education, working status, geographical location, reading habits, and marriage preferences play a significant role in early sexual initiation among female adolescents (6). Although adolescents acquire knowledge about HIV/STI prevention through various methods, the rates of HIV/STIs remain high, particularly in low-income countries (7). Knowledge of the consequences of risky sexual behaviors does not influence adolescents’ sexual decision-making (7). The literature documents that exposure to sexually explicit Internet material (SEIM) is a significant factor influencing adolescent males’ engagement in sexual activities, and regular usage of SEIM leads to online pornography consumption (8). These adolescents tend to learn sex education through SEIM, which often involves misinformation and inadequate knowledge (8). On the other hand, adolescent females tend to gain knowledge from their peers and they are more likely to initiate sexual activity to fit in with their peers (9).

New HIV infection rates in Cambodia were reduced from 1.3% to 0.6% in the last two decades (National Center for HIV/AIDS, Dermatology, and STD [NCHADS], 2022), however, unintended pregnancy rates remained high as 9% among women aged 15–19 years (National Institute of Statistics, 2022). In addition, 18% of women and 5% of men aged 15–49 years experienced STIs or STI symptoms (National Institute of Statistics, 2022). The biggest challenges identified in the literature were a lack of formal sexual education and limited access to health care services on this topic (10).

The principle of Khmer culture is known as *Chbab Srey* which emphasizes women's obedience and respect their husband (11). Traditionally, Cambodian culture viewed sexuality as taboo and premarital sex was forbidden (11). Women's virginity until marriage was expected for their future husband while men enjoyed freedom to have sex (12). Sex was considered women's duty for their husband while having sex was a men's pleasure (13). These cultural norms have changed slowly among Cambodian adolescents with the influx of the western culture. A new dating trend toward causal relationship was noted among them (10). They met through social media or friends and developed intimate relationships often led to engage in sexual intercourse (10). However, many of these adolescents expressed that they did not have proper sexual education focusing on safe, responsible, and consensual relationship (14), yet it is not clear what motivates these adolescents to engage in sexual activities. It is important to find how adolescents make sexual decisions to provide effective interventions to promote sexual and reproductive health. This study explored the internal and external decision-making factors that influence Cambodian adolescents’ engagement in sexual activity using a qualitative method to understand this population and develop appropriate educational interventions to minimize the negative outcomes of early sexual engagement.

## 2 Methods

### 2.1 Study design, participants, and recruitment

This study was conducted in accordance with the Standards for Reporting Qualitative Research (SRQR) (15), ensuring adherence to established guidelines for qualitative research methods and reporting. We adopted a descriptive qualitative methodology to understand the sexual decision-making processes among Cambodian adolescents. Eligibility for participation required individuals to be single, have a history of sexual encounters, and be willing to openly discuss their views and experiences related to the research themes. The study focused on Cambodian adolescents, due to the increasing concern over sexual health and decision-making in this age group. Adolescents in Cambodia are at a critical juncture in terms of sexual development and face significant barriers to accessing accurate sexual health information, which may lead to risky behaviors. Focusing on adolescents who are single but sexually experienced allows us to explore the complexities of sexual decision-making in a context where traditional norms may clash with modern influences, providing valuable insights into the specific challenges faced by this demographic in a rapidly changing social environment.

Participants were recruited using a combination of convenience and snowball sampling methods to ensure diversity and openness in sharing sensitive information. The decision to use these methods was informed by the sensitive nature of the research topic and the anticipated challenge of openly discussing sexual behaviors among Cambodian adolescents. Initially, potential participants were identified through various channels, including schools, social networks, and online communities based in Phnom Penh. We began by disseminating information about the study through platforms such as Facebook and Instagram. The recruitment message included details about the purpose of the research, the voluntary nature of participation, and assurances of confidentiality and privacy during the interview process. Once individuals expressed interest, they underwent an initial screening with a recruiter to ensure they met the inclusion criteria (i.e., being single, sexually experienced, and open to discussing sexual decision-making). Basic demographic information, such as age and gender, was collected during this stage to confirm eligibility. After screening, the participants were scheduled for interviews. Participants who had already completed the interview were asked to refer others within their social circles who might meet the study's criteria and be willing to discuss their sexual experiences. This approach was chosen because it allowed participants to feel more comfortable knowing that their peers had already engaged in the study, which built trust and encouraged participation.

### 2.2 Data collection

Informed consent was obtained directly from all participants before conducting interviews. None of the participants were under the age of 16. They were informed about the study's purpose, the voluntary nature of participation, confidentiality, and their right to withdraw at any time.

The interviews, conducted in Khmer, were open-ended and semi-structured, designed to encourage participants to reflect on and discuss their sexual decision-making experiences. The interview began with the question “What factors do you think influence your decision to have sex?” and expanded to include the impact of friends, parents, schools, local communities/neighbors, cultural beliefs or attitudes, and government on sexual decisions. MRTS Consulting Ltd., a Cambodian company with more than 15 years of experience in qualitative research, facilitated the interviews. Although the company does not have direct experience conducting interviews specifically on topics related to sexual or reproductive health, they have extensive experience handling sensitive topics in other domains, such as marketing research and consumer behavior. Prior to fieldwork, the lead researcher conducted three training workshops for the research team covering study objectives, materials, expected outcomes, and interview techniques. The research tools, including questionnaires and demographic queries, were pre-tested to ensure effective data collection. This included verifying participants’ comprehension, appropriate interview length, and whether any questions needed to be added or removed. Special emphasis was placed on preparing interviewers to handle sensitive topics related to sexual decision-making. This included role-playing exercises, discussions on maintaining a neutral and non-judgmental tone, and techniques for ensuring participant comfort and confidentiality. Interviewers were also trained in ethical guidelines for discussing sensitive subjects and ensuring that participants were not pressured to continue discussing any topic they felt uncomfortable with. If a participant chose not to respond, interviewers were instructed to respect this decision without further prompting. Gender-matched interviewers were assigned to promote open and honest dialogue, with only the participant and interviewer present during the sessions.

The in-depth interviews were conducted between March 20 and June 30, 2022. The recorded sessions, held in person and via Zoom (with video turn-off options), were securely stored on a password-protected server with restricted access. Confidentiality agreements were signed by all team members, safeguarding participant privacy. Participants received a US$30 incentive in sealed envelopes or via bank transfer for Zoom interviews. This study was ethically approved by the Institutional Human Subjects Review Board of X University.

### 2.3 Data management and analysis

The interviews, initially conducted in Khmer, were translated and transcribed directly into English by a skilled transcriber. To ensure the integrity of the transcribed content, the research team verified 20% of the transcripts, focusing on accuracy of content, language, and grammar. A validation step involved a subset of interviewees (two males and one female) to confirm the fidelity of the transcripts. These participants reviewed their interview transcripts with our moderators/interviewers in Khmer, affirming the authenticity of the transcribed content.

For data analysis, we utilized thematic analysis according to Braun and Clarke's framework (16). Two members of the research team undertook the coding process. After an in-depth review of the English transcripts to familiarize themselves with the data, the initial codes were generated. These codes were then categorized into emerging themes. A thematic “map” or coding tree was developed to align potential themes with coded excerpts and the entire dataset, ensuring themes were directly derived from the data. Themes were defined and named to highlight their distinct features, and a narrative analysis was constructed, linking the themes to the research questions and existing literature. An external researcher, not involved in the initial analysis, cross-verified the data to ensure consistency with the findings.

## 3 Results

The study included 30 Cambodian adolescents, split evenly between genders with 15 males and 15 females. The average age was approximately 18 years, and all the participants were Buddhists. The majority (27 out of 30) consumed alcohol, whereas 6 were smokers. They had average pocket money of 248 USD. In terms of sexual experiences, the average age at the first sexual encounter was around 17 years, and the number of sexual intercourse experiences varied among participants, with several reporting more than five times.

The results of the data analysis revealed that both the internal and external factors influenced the decision-making process. Internal factors included SEIM and arousal from sexy outfits, while external factors included foreign vs. Khmer culture, the surrounding environment including community, peers, and family, and educational advice received at school. Descriptions of these factors and representative quotes are as follows.

### 3.1 Internal factors

#### 3.1.1 Influences of SEIM

Approximately two-thirds of the male participants encountered sexually stimulating media. Some participants watched pornography and were exposed to less stimulating content through social media or the Internet. All who said they watched porn were males, and a woman responded that she saw sexual content on the Internet by accident. “I didn't intend to watch. I accidentally found it on the internet, so I watched some parts of it. I didn't watch the whole movie” (Female, aged 19). The frequency of watching porn ranged from a few times to every day, and the reasons for watching porn were curiosity, ways to relieve stress, and influence of other people around them. “They *(friends) told me about that (porn) and they convinced me to give it a try... They told me that it's the best feeling ever and I should have tried it” (Male, aged 19).* Many participants said that sexual desire had the greatest influence. “*It's because of my age. I have grown up, so I have become curious... The sexual desire because I have reached a point of life where I become curious of many things” (Male, aged 18).* They claimed that pornography had become a vehicle for sexual excitement. One participant described the impact of pornography on the decision to have sex as significant. “*Great influence because it provokes my sexual desire. It's like a bull in rut. All I think of is having sex” (Male, aged 16)*.

#### 3.1.2 Arousal from sexy outfits

Participants said that they were exposed to sexy outfits through social media, service ladies at KTV, and nearby women or their girlfriends. Only male participants responded that they felt horny about sexy outfits, which was 60% of all male participants. They keep staring at someone in sexy clothes and feel sexually aroused, thinking they want to have or imagine having sex with her. One participant expressed his thoughts when he saw people wearing sexy outfits.

I have looked at them. It turns me on to see them in sexy outfits. I am thinking of the time I could sleep with her. How great would it be if I could sleep with her. (Male, aged 19)

### 3.2 External factors

#### 3.2.1 Foreign vs. Khmer culture

Most participants considered Khmer culture boring and strict in terms of sexuality. This is because Khmer culture requires women to be virgins and does not allow pre-marriage sex or homosexuality. One participant argued that the relatively sexually closed Khmer culture should be changed.

It's wrong to do that while studying. It's more open in the US and I think it's good that way. I want some changes in our culture. They should be free to do anything they want and they can still go to school. School in our country tends to not accept students who had sex while studying. They are concerned about their school credit. They discriminate against the students which affect their future. (Female, aged 18)

On the other hand, they described foreign cultures, such as Korea, Japan, the US, Vietnam, China, and Thailand, as wearing revealing clothes or no bras, having several partners, guaranteeing rights and freedom to sex, having no shame or fear of sex, and being confident. They mentioned that Khmer culture is gradually changing open in the process of acquiring foreign culture. Approximately one-third of the participants affirmed the impact of the influx of foreign cultures on sex decisions. One participant claimed that the influx of foreign culture had a social influence.

There is more freedom and the influx of foreign culture. We are less shy. European culture focuses on emotional connection. This culture also influences us to be more decisive in doing it by observing the surroundings. We tend to think that it’s fine to do it because everyone around us is doing it, too. (Male, aged 17)

They said that foreigners are easily seen on the street or in pornography and that their culture has a great influence on sex decisions. In particular, they mentioned the influence of the kiss and hug culture and sexy and revealing clothes.

The way they (foreigners) greet each other and behave is very inviting. It can turn me on. They always wear sexy and revealing clothes that can turn us on. (Male, aged 17)

#### 3.2.2 Surrounding environment: community, peers, and family

Most participants answered that they were influenced by people around them. They were motivated on sexual activity by various people they meet in their everyday lives.

About half of the participants said they were influenced by people who touch each other in public places. In particular, they talked about their experiences of seeing people in physical contact in public and agreed on the impact of this experience. One participant shared her experiences and thoughts of witnessing someone who looked intimate in public.

I see people kissing each other and it makes me excited. They are around my age or maybe one year older than me. (Female, aged 19)

Neighbors also wielded a strong influence on adolescents through stories. They deliberately or unintentionally shared stories about their sexual experiences, and the participants who heard them said they had become curious about having sex. Influential neighbors were mostly peers and seniors.

Elder people are talking with their friends but they don't notice young people are around. We, as young people, become curious about that. They are not likely to do it when they are under full control of their parents but they will choose to do it later if they have the chance. They will find their own partner to actually practice the knowledge that they have learned so far. They have heard about that from the neighbors. Young people are not very considerate sometimes. They tend to follow this guy or that guy and they don't see any issue with doing that fun thing. (Male, aged 17)

They could hear vivid experiences, but they also received advice about incorrect sexual knowledge from their neighbors.

They said that it feels good to do it and they told me not to use a condom. Sex without condom is easier. I was at a coffee shop with them and they were telling me about it. (Male, aged 18)

Two-thirds of the male participants affirmed the influence of their parents on their sex decisions. They said they wanted to have a partner when they saw their parents looking happy. They were looking for a suitable partner to build a happy family, with their parents as their role models. As such, the intimacy of their parents, which adolescents always watch, influenced their dreams of an ideal family.

I observe how intimate they are. I imagine that I might have such happiness one day when I get married to my partner. They are very intimate and they love each other. So, I start looking for a suitable girlfriend. (Male, aged 19)

The influence of friends was emphasized more by male participants. Their friends shared their sexual experiences and pleasures, which motivated them to have sex. On the other hand, female participants answered that they were influenced by the ideal appearance of their friends and partners.

They talk about having sex and emphasize that it's the best feeling ever. It makes me want to try that. (Male, aged 19)

#### 3.2.3 Educational advice at school

According to the participants, sex education for Cambodian teenagers is mainly provided in science classes, such as biology. They responded that school teachers provide information related to sex, such as reproductive health, hormones, STIs, puberty, contraceptive measures, and consequences that may be caused by sex. Some teachers make them feel positive about sex through sex education.

It's from biology class. Teacher talks openly about reproduction health and they also educate us about safe sex. Normally, teacher raises examples related to sexual health and intercourse as well. To some extent, it provokes the drive of the young student to think of having done it once. Teacher even jokes around about having saying that if you want to know how it feels like, you have to practice. We wish to do it one day if there's an opportunity. (Male, aged 19)

However, some teachers discourage students from having sexual experiences and advise them to study.

They give us advice and they tell us not to be involved in such things yet because we are still young. (Male, aged 18)

## 4 Discussion

This study contributes to understanding the sexual decision-making process among Cambodian adolescents. The findings reveal several internal and external factors influencing their choices. This study reveals a notable gender difference in the intentional consumption of pornography, with all male participants reporting intentional exposure. This aligns with existing research that often indicates higher rates of deliberate engagement with SEIM among male adolescents (8, 17). Most males in this study reported watching pornography intentionally, driven by curiosity, stress relief, and peer pressure. The frequency of pornography consumption varied, with some participants citing it as a significant factor driving their sexual desires. A systematic review of 19 studies established a link between the consumption of pornography and a range of behavioral, psychophysical, and social consequences (18). Notably, these consequences include initiating sexual activity at an earlier age, engaging with multiple and/or casual partners, and replicating risky sexual behaviors (18). Adolescents exposed to SEIM typically have an earlier sexual debut, confirming the role of pornography in accelerating sexual activity and increasing the propensity for casual sex encounters (19, 20). In contrast, females predominantly reported accidental exposure to sexually explicit content. This distinction highlights potential differences in the sources and motivations for sexual stimuli between males and females. It underscores the importance of investigating how unintentional exposure affects female adolescents’ perceptions of sexuality. Additionally, there is a pressing need for comprehensive sex education, especially for male adolescents, considering that 97.9% of Cambodian adolescents use mobile phones, which often serve as mediums for accessing explicit material (21).

This study indicated that only male participants expressed feeling sexually aroused when observing individuals in sexy outfits. This aligns with the traditional gender norms and expectations surrounding visual stimuli and male sexuality. The tendency of males to experience increased arousal and engage in sexual behavior in response to sexy outfits can be theoretically grounded in the S-O-R model (Stimulus-Organism-Response) (22). This model suggests that sexy outfits act as a stimulus (S), initiating cognitive and affective processing (O) in males, which then leads to a heightened inclination toward sexual behavior (R) (22). Female participants did not report feeling aroused by sexy outfits, indicating a potential difference in the nature of the stimuli influencing sexual desire.

The influence of cultural norms on sexual decision-making is evident in how participants view Khmer culture as conservative and restrictive. Many participants desired a more liberal approach to sexuality, referencing the openness and acceptance seen in foreign cultures, such as the US, Korea, and Japan. Males particularly highlighted the liberality of these cultures, whereas females often noted aspects such as more revealing clothing styles. Similarly, Indian television advertisements have been found to influence the social and cultural values of Pakistani youth, affecting their attitudes towards sexuality, gender roles, dressing styles, and language use (23). Additionally, exposure to Internet media, notably pornography, impacts perceptions on sex. For instance, Chinese youth exposed to Western pornography were more likely permissive toward sex (24). This attitude was associated with poor mental health outcome as these adolescents had less satisfied with their lives (24). This showcases the broader impact of globalization, especially how the media and the Internet from one culture can significantly influence the values and behaviors of another. In Cambodia, the influence of globalization, particularly through exposure to foreign media and practices, underscores the need for culturally sensitive sex education that addresses these changing societal norms (25). The perceived conservatism of Khmer culture, particularly in terms of premarital sex and homosexuality, suggests a cultural tension between traditional beliefs and modern influences (25). Adolescents’ desire for a more open cultural approach to sexuality may reflect the impact of globalization and exposure to Western cultures, a phenomenon observed in various Asian contexts (26). This cultural shift has significant implications for sexual health education and policies, as it calls for a balance between respecting cultural norms and addressing the evolving sexual health needs of adolescents.

The findings of this study, which explore the decision-making process regarding sexual activity among adolescents, resonate with the existing literature on the influence of the surrounding environment and social interactions on adolescent sexual behavior (13, 27). The participants’ experiences of being influenced by public displays of affection align with broader observations of the impact of sociocultural factors on adolescent sexuality. Adolescents are influenced by witnessing physical intimacy in public spaces. This suggests a global trend where exposure to more open expressions of sexuality, possibly through globalization and the media, shapes adolescents’ perceptions and behaviors. The narratives shared by older community members can spark curiosity and influence decision-making among adolescents, reflecting a pattern of informal sex education (13, 27).

Notably, male adolescents are predominantly influenced by their peers’ sexual experiences. The study by Junqiang Dai et al. (28) provided critical insights into the neural underpinnings of peer influence susceptibility and risk-taking behaviors in adolescents, offering a valuable perspective for understanding the dynamics of peer influence in adolescent sexual decision-making. The findings suggested that the neural responses in the nucleus accumbens, a key brain region associated with reward processing, were significantly linked to adolescents’ susceptibility to peer influence and their propensity for risk-taking behaviors (28). In contrast, female adolescents reported being influenced by the idealized appearance of their friends’ partners. This suggests that, for female adolescents, the physical attractiveness and aesthetic qualities of a potential partner play a significant role in their sexual decision-making process. Understanding this aspect of peer influence is crucial for developing effective sexual and relational education programs. This calls for a more nuanced approach that addresses the diverse factors influencing adolescent sexual behavior, including the impact of societal beauty standards and the role of physical appearance in relationship dynamics.

The findings of this study revealed intriguing gender differences in how family dynamics influence adolescent sexual decision-making. Notably, male participants reported being significantly influenced by observing their parents’ intimacy, which was associated with their aspiration for a happy family life. In contrast, this study did not find significant parental influence among female participants. Females may be more influenced by peer relationships and the media than family dynamics in sexual decision-making (9). The results of this study highlight the importance of family dynamics, particularly parental relationships, in shaping male adolescents’ sexual aspirations and decision-making. According to the family communication patterns theory, the nature and quality of family communication profoundly impact adolescents’ sexual self-efficacy and their ability to communicate about sex with their partners (29). This highlights the need for further research on how different family dynamics and parental models differently impact male and female adolescents’ approaches to sexual relationships.

Participants of both genders reported receiving sex education at school, predominantly through biology classes. While some educators provide extensive information on reproductive health and safe sex practices, others tend to discourage sexual activity among students. In Cambodia, sex education is largely focused on health and suffers from limited availability and scope, often lacking separate health education classes (14). Thus, there is a growing need for a more comprehensive, accessible, and culturally sensitive approach to sex education in Cambodia. Emphasizing the importance of digital platforms and the involvement of various stakeholders, such as schools, parents, healthcare professionals, and government agencies, in delivering holistic sex education programs is crucial (30). For example, the Netherlands employs digital platforms like “Sense.info” to provide both professional and user-generated content on sexual information. This approach is particularly effective for youth who have greater sexual knowledge, experience, and challenges, as well as for those who engage more in discussions about sex with their peers (31).

## 5 Conclusion

In conclusion, the sexual decision-making process among Cambodian adolescents is complex and is influenced by a combination of internal and external factors. This study highlights the impact of explicit online content, visual stimuli, cultural norms, peer influences, and educational advice on adolescents’ choices. To promote healthy sexual decision-making, there is a need for comprehensive and culturally sensitive sex education that addresses the diverse influences on adolescents. Understanding these variations can contribute to more effective and inclusive approaches to sex education and health promotion.

## 6 Limitations and strengths

The strengths and limitations of this study are as follows. While the mechanisms influencing adolescents’ sexual decision-making could be more complex and varied, a limitation of this study is that it primarily included urban residents, leaving the experiences of rural adolescents underrepresented. This remains a topic for future research. A notable strength is our ability to recruit female students who are typically less open about sexual issues, making up half of the participants, allowing for a thorough exploration of both male and female experiences. Another strength lies in the rigorous conduct of the study, managed and analyzed by an experienced and well-trained research team.

## Data Availability

The original contributions presented in the study are included in the article/Supplementary Material, further inquiries can be directed to the corresponding author.
